# Fighting female genital mutilation/cutting (FGM/C): towards the endgame and beyond

**DOI:** 10.1186/s12978-023-01601-3

**Published:** 2023-03-29

**Authors:** Saidou Sabi Boun, Akaninyene Otu, Sanni Yaya

**Affiliations:** 1 School of International Development and Global Studies, Faculty of Social Sciences, University of Ottawa, 120 University Private, Ottawa, ON K1N 6N5 Canada; 2 Department of Infection, Hull University Teaching Hospitals NHS Trust, Hull, UK; 3 The George Institute for Global Health, Imperial College London, London, UK

**Keywords:** Female genital mutilation, Female genital cutting, Female circumcision, Reproductive health, Sexual and reproductive health and rights (SRHR)

## Abstract

Despite the criminalization of the practice by numerous laws and international treaties in most countries concerned, female genital mutilation/cutting (FGM/C), although on the decline overall, is stagnating or tending to increase in some parts Africa. This relative failure in the fight against FGM/C could be explained from an institutional perspective. Although these struggles affect the regulatory mechanisms, which include laws, they hardly touch the normative mechanisms, which constitute the set of values deemed socially acceptable by a society, and the cultural and cognitive mechanisms, which are the manifestations of the ideologies or beliefs of a group. The naming of FGM/C among certain ethnic groups, which is part of the normative character of the social institution, rather valorizes them and makes uncut girls/women feel "dirty" or "unfit”. In these communities, women who have undergone FGM/C are viewed by society as women of honour while uncut girls are perceived as promiscuous and victims of mockery, rejection, or exclusion by the community. In addition, since excision ceremonies and rituals are exclusively reserved for women, many see them as a way of freeing themselves from the rules of patriarchy and male domination that are omnipresent in the societies concerned. Informal mechanisms such as the use of witchcraft, gossip, and beliefs related to the supernatural power of the excisors underpin the cultural-cognitive nature of FGM/C practice. As a result, many families are reluctant to challenge the cutters. The fight against FGM/C can be more effective by addressing the normative and cultural-cognitive roots that form the basis for its perpetuation. This can be achieved by avoiding moralizing the practice, involving those who resist the practice in a context of high prevalence, known as "positive deviants," and using productive methods from the societies concerned. This will create a social environment in which FGM/C is increasingly perceived as less favourable and will ultimately allow for a gradual reform of the normative and cultural-cognitive character of societies that practice FGM/C. Education of women and social mobilisation are critical tools which can act as powerful levers in shifting attitudes about FGM/C.

## Introduction

Despite the global recognition of female genital mutilation/cutting (FGM/C) as a gross violation of girls and women's human rights and health, this harmful practice persists [1]. FGM/C refers to any non-therapeutic procedure that involves partial or total removal of a woman's external genitalia [2]. The United Nations Population Fund (UNFPA) estimates that 4.3 million girls will be at risk of FGM/C in 2023, and the number is projected to reach 4.6 million by 2030 [3]. Rooted in gender inequality and power imbalances, FGM/C is now recognized as an extreme form of gender-based violence that distorts girls’ bodies and endangers their lives with no health benefits for them. However, the gender and social norms that underpin this damaging practice still have a strong influence despite attempts spanning nearly a century to eliminate it, and this has been further exacerbated by factors such as conflict and rising poverty and inequality.

The World Health Organization (WHO) states that over 200 million women worldwide have undergone FGM/C—many before age 15. Among the 30 countries affected by FGM/C, 27 are in Africa, with prevalence rates of women aged 15–49 years who have undergone mutilation equal to 98% in Somalia, 97% in Guinea and 93% in Djibouti [4]. FGM/C has no benefits but rather has adverse health consequences for women, which can be immediate (hemorrhage, infections such as HIV, urinary retention, and shock that can lead to death). In the long term, mutilated women may experience urinary difficulties, vaginal and menstrual problems, risk of complications during childbirth, and psychological issues such as anxiety, depression, and post-traumatic stress [2, 5]. Age, area and region of residency, religious affiliation, educational status, and household wealth have been identified as significant predictors of FGM/C [1, 6].

Despite the criminalization of FGM/C, which has led to an overall, albeit slow, reduction in its practice worldwide, it is reportedly stagnating or even increasing in some regions of Africa. The prevalence of FGM/C in Guinea rose from 96 to 97% between 2005 and 2012 for women of childbearing age [7], and most women/girls favoured its perpetration. From 2005 to 2012, girls and women attending FGM/E practices in Guinea increased from 69 to 76% [7].

### From education to medicalization, the evolution of the fight against FGM/C

In a bid to accelerate progress against FGM/C, education has been widely adopted as a critical approach to increase awareness of the dangers of FGM and foster questioning of the social norms which promote the practice of FGM/C [8]. The assumption has been that communities would be motivated to abandon FGM/C if they knew the risks involved [9]. Socio-demographic survey data, an approach inspired by dependency theories, suggested that traditional societies naturally evolve towards modernity by abandoning FGM/C due to education and urbanization [5]. Unfortunately, data shows that communities of all socioeconomic classes still practice FGM/C, despite being aware of its harmful effects because of its social and cultural importance [10]. Also, health education campaigns have produced unexpected secondary implications, such as increased medicalization of FGM/C, as communities have become more aware of the health risks [11]. The medicalization of FGM/C refers to situations in which it is performed by health personnel, in a private or public hospital/clinic, at home or elsewhere, at any stage of the woman's life with reduced severity of cutting [12].

In the 1990s, faced with the relative failure of the educational approach and modernization theories, Northern feminists tried to situate the struggle within the framework of the oppression of Black women in a patriarchal system [5]. This vision was denounced by postcolonial feminists who viewed it as a form of neo-colonialism, reopening a more ancient debate between relativism and universalism of cultural values [9].

The movement against FGM/C then put forward the framework of human rights, children's rights, and women's protection to advocate the prohibition of the practice. The United Nations General Assembly ratified this (UNGA), adopting a Resolution to ban female genital mutilations worldwide, "whether committed within or outside a medical institution," in December 2012 [A/RES/67/146]. This was further supported by the United Nations Commission on the Status of Women's 57th session in 2013, which focused on violence against women and girls, including FGM/C. A zero-tolerance policy for FGM/C has been endorsed by the WHO, promoting the rejection of all forms of FGM/C, including the least invasive ones, especially those performed in hospitals, regardless of the age of the girl/woman [12]. This zero-tolerance policy has since been reaffirmed and re-adopted by many other international health and development organizations [12]. Some authors consider medicalization a harm-reduction method for FGM/C, arguing that it mitigates acute complications by as much as 70% using aseptic techniques and anaesthetics [11, 13]. Parents have adopted the medicalization of FGM/C (done with a small incision) to protect their daughters from more invasive forms [14]. Even though their opposition to the medicalization of FGM/C, the United Nations Children's Fund (UNICEF) estimates that ¼ of the women who have undergone FGM/C worldwide, or approximately 52 million, were performed by health personnel [15].

### The relative failure of the fight against FGM and the institutional iceberg

This relative failure to combat FGM/C effectively in African countries could instead be analyzed from an institutionalist perspective. Quoted by Benoit Prevost, North Douglas defines institutions as: "Constraints established by men that structure human interactions. They consist of formal constraints (such as rules, laws, constitutions), informal constraints (such as norms of behaviour, conventions, imposed codes of conduct) and the characteristics of their application" [16].

Thus, all social groups have rules of play that differ in size, shape, and context. There is, therefore, no blank slate on which to write scenarios for change without considering what already exists [17]. According to this idea, to change a society, to change the rules of the game of a community and to reform the institutional constraints that weigh on it, it would be necessary to improve, at the same time, the regulatory mechanisms. These include the laws that reward or punish a person in response to their choices. It would also be expedient to reform the normative mechanisms, which are the set of values deemed socially acceptable by society and to improve the socio-cultural mechanisms which are the cultural and cognitive manifestations of the ideologies or beliefs of a group such as religious dogmas. Any institution is thus at the interaction of these three characters. The interaction of these three traits shapes the theoretical framework within which individuals see, make choices and interpret the world [17].

It is the regulatory character that is the most visible part of the institutional iceberg of a society. Composed of laws and established practices, it allows control the community by the power of rewards or punishment. The normative character will enable it to understand the directive mechanisms, the values of things, and the socially acceptable practices [17]. Appropriate behaviours can influence people during a transaction by appealing to honour or shame. It is the first invisible part of the iceberg of an institution. Cognitive, cultural elements are cultural and cognitive manifestations reflected in textual ideologies, in symbols of group affiliation and belief such as religion, nationality or language. This is the second invisible part of the iceberg of an institution. These elements combine to form the rules of play of an institution in a society [17].

FGM/C's perpetuation could be viewed as being driven by the interaction of regulatory, normative, and cultural-cognitive mechanisms. Thus, although most countries affected by the practice have reformed the regulatory mechanisms through ratifying laws prohibiting and penalizing FGM/C, the reforms have had little impact on the normative and cultural-cognitive characteristics of the practice. For example, in Guinea, among the Guerze, the term used to designate FGM/C translates as "washing," while among the Peul and Soussou, the expression can be translated as "dressing" [7]. This way of referring to FGM/C, which is based on the normative nature of the social institution, rather valorizes female genital mutilation, and makes uncut girls/women feel "dirty" or "unfit." This view is further reinforced by the idea that FGM/C is part of traditional heritage, and renouncing it would be tantamount to abandoning its cultural values [18]. In many communities practicing FGM/C, women who have undergone FGM/C are viewed as women of honour, unlike uncut girls, who are perceived as promiscuous. The latter are often the victims of mockery, rejection or exclusion by the community [18]. In addition, because FGM/C ceremonies and rituals are reserved exclusively for women, many women see them as a space to free themselves from the rules of patriarchy and male domination that are omnipresent in the societies concerned [7, 18].

Informal mechanisms such as the use of witchcraft, gossip, and beliefs related to the supernatural power of the exercisor underpin the cultural cognitive nature of FGM/C practice. As a result, many families are reluctant to contradict the excisors [7]. Thus, the intertwining of these three characteristics of the social institution has led to an explosion of medicalized FGM/C, mainly because many families are now aware of its harmful effects [5]. Indeed, health professionals are increasingly performing medicalized FGM/C, with a vision of "harm reduction" (reducing health risks for girls), for personal gain or to respond to demands and pressures from the communities [19]. For example, in Guinea, the proportion of FGM/C performed by health professionals increased from 15 to 31% between 2005 and 2012 [7]. In Egypt, an estimated 80% of women who are mutilated are mutilated by a health professional [12]. Consequently, the logic of FGM/C practice which is rooted in normative, cultural, and cognitive character still dominates the societies affected by the practice. However, the regulatory character could have had deterrent effects [20]. The persistence of normative and cultural-cognitive characteristics could explain, for example, why some excisors are seldom prosecuted [7].

### Avenues for sustainable solutions

The fight against FGM/C can be made more effective by addressing the normative and cultural-cognitive roots that form the basis for its perpetuation. This can be achieved by avoiding moralizing about the practice, involving people who resist the practice in a context of high prevalence called "positive deviants," and using productive methods emerging from the societies involved. This will create a social environment in which FGM/C is increasingly seen as less favourable and ultimately allow for a gradual reform of the normative and cultural-cognitive character of societies that practice FGM/C. A study conducted in Guinea showed that families who refused to have their daughters cut either had a social support network that shared the same values as them or was economically independent of the traditional solidarity network [14].

Endogenous solutions have been successfully developed in Benin and Kenya which respected the communities' traditions and consisted of implementing rituals without cutting [21]. These are identical to traditional ceremonies, which value the customs and the excisors but without genital mutilation.

Education remains a critical tool for fighting FGM/C. Research has shown that girls and women with a primary education are 30% more likely than those with no education to oppose FGM/C and this rises to 70% among girls and women with a secondary education or higher. FGM/C is substantially less prevalent as a mother’s educational level rises. Girls whose mothers have a primary education are 40% less likely to undergo FGM/C than those whose mothers have no education [22]. Therefore, education of girls and women and improvement of their livelihood opportunities should be given greater priority. Efforts to raise awareness about the dangers of FGM/C in communities should be strengthened by leveraging social media and cultural/religious groups to propagate the key messages [23]. The topic of FGM/C should be introduced into the education curricula at all levels and public fora. All these will act as powerful levers in shifting attitudes about FGM/C.

## Conclusion

FGM/C is a complex problem which impacts the rights of women and girls with untoward consequences for their health, education, and income. Target 5.3 of the Sustainable Development Goals aims to eliminate all harmful practices, such as child, early and forced marriage and female genital mutilations by 2030. Collective and well-funded action is urgently required across a diverse group of stakeholders to address the normative and cultural-cognitive roots of FGM/C, prioritize education of girls/women and strengthen social mobilization. Only then will this shared goal of ending FGM become a reality.

## Data Availability

Not applicable.

## References

[1] Yaya S, Ghose B (2018). Female genital mutilation in Nigeria: a persisting challenge for women’s rights. Soc Sci.

[2] World Health Organization. Female genital mutilation (fact sheet) [Internet]. 2023 [cité 28 janv 2023]. Available at: https://www.who.int/news-room/fact-sheets/detail/female-genital-mutilation.

[3] United Nations Children’s Fund (UNICEF). World will miss target of ending FGM by 2030 without urgent action-including from men and boys [Internet]. 2023 [cité 14 mars 2023]. Available at: https://www.unicef.org.uk/press-releases/this-year-4-3-million-girls-are-at-risk-of-female-genital-mutilation-according-to-the-latest-unfpa-estimates/.

[4] United Nations Children’s Fund (UNICEF). Female Genital Mutilation/cutting: a Global Concern. UNICEF’s Data Work on FGM/C. Unicef; 2016.

[5] Andro A, Lesclingand M (2016). Les mutilations génitales féminines. État des lieux et des connaissances. Population.

[6] Ahinkorah BO, Hagan JE, Ameyaw EK, Seidu AA, Budu E, Sambah F (2020). Socio-economic and demographic determinants of female genital mutilation in sub-Saharan Africa: analysis of data from demographic and health surveys. Reprod Health.

[7] Haut-commissariat des Nations Unies pour les droits de l’homme. Rapport sur les droits humains et la pratique des mutilations génitales féminines/excision en Guinée [Internet]. République Guinée; 2016 [cité 28 févr 2023]. 32. Available at: https://www.ohchr.org/Documents/Countries/GN/ReportGenitalMutilationGuinea_FR.pdf.

[8] Ameyaw EK, Yaya S, Seidu AA, Ahinkorah BO, Baatiema L, Njue C (2020). Do educated women in Sierra Leone support discontinuation of female genital mutilation/cutting? Evidence from the 2013 Demographic and Health Survey. Reprod Health.

[9] Hernlund Y, Shell-Duncan B (2007). 1. Transcultural positions: negotiating rights and culture. Transcultural bodies.

[10] Shell-Duncan B (2008). From health to human rights: female genital cutting and the politics of intervention. Am Anthropol.

[11] Pearce AJ, Bewley S (2014). Medicalization of female genital mutilation. Harm reduction or unethical?. Obstet Gynaecol Reprod Med..

[12] Organisation mondiale de la Santé (2011). Stratégie mondiale visant à empêcher le personnel de santé de pratiquer des mutilations sexuelles féminines. Droit Déontolog Soin.

[13] Shell-Duncan B (2001). The medicalization of female “circumcision”: harm reduction or promotion of a dangerous practice?. Soc Sci Med.

[14] Doucet MH, Delamou A, Manet H, Groleau D (2020). Correction to: Au-delà de la volonté: les conditions d’empowerment nécessaires pour abandonner les mutilations génitales féminines à Conakry (Guinée), une ethnographie focalisée. Reprod Health.

[15] United Nations Children’s Fund (UNICEF). Female Genital Mutilation: A New Generation Calls for Ending an Old Practice [Internet]. New York: Unicef; 2020. 8. Available at: https://data.unicef.org/resources/female-genital-mutilation-a-new-generation-calls-for-ending-an-old-practice/.

[16] Prevost B. Douglass North : hétérodoxie néo-institutionnelle versus néolibéralisme ?: Éléments pour un débat sur les réformes institutionnelles dans les PVD. Rev Régulation [Internet]. 16 févr 2010 [cité 20 nov 2022];(7). Available at: http://journals.openedition.org/regulation/7719.

[17] Andrews M. The Limits of Institutional Reform in Development: Changing Rules for Realistic Solutions [Internet]. 1^re^ éd. Cambridge University Press; 2013 [cité 9 déc 2022]. Available at: https://www.cambridge.org/core/product/identifier/9781139060974/type/book.

[18] Behrendt A. Traditions et droits. L’excision en Afrique de l’ouest. Plan Int Dakar Sénégal [Internet]. 2006; Available at: http://www.strategiesconcertees-mgf.be/wp-content/uploads/plan-excision-en-afrique-de-louest-2006.pdf.

[19] Doucet MH, Pallitto C, Groleau D (2017). Understanding the motivations of health-care providers in performing female genital mutilation: an integrative review of the literature. Reprod Health.

[20] Shell-Duncan B, Hernlund Y, Wander K, Moreau A. Legislating Change? Responses to criminalizing female genital cutting in Senegal. 10.1111/lasr.12044 PMC3997264 24771947

[21] Finke E (2006). Genital mutilation as an expression of power structures: ending FGM through education, empowerment of women and removal of taboos. Afr J Reprod Health.

[22] United Nations Children’s Fund (UNICEF). The Power of Education to End Female Genital Mutilation. New York: UNICEF; 2022.

[23] Ameyaw EK, Anjorin S, Ahinkorah BO, Seidu AA, Uthman OA, Keetile M (2021). Women’s empowerment and female genital mutilation intention for daughters in Sierra Leone: a multilevel analysis. BMC Womens Health.

